# Assessment of the clinical efficacy of cell-assisted lipotransfer and conventional fat graft: a meta-analysis based on case-control studies

**DOI:** 10.1186/s13018-017-0645-5

**Published:** 2017-10-19

**Authors:** Yu Wang, Yanfei Wu

**Affiliations:** 1 Department of General Surgery, The No. 1 Hospital of Jiaxing City, Jiaxing, 314001 Zhejiang Province People’s Republic of China; 2 Jiaxing University College of Foreign Studies, No.56 Yuexiu Road (South), Jiaxing, 314001 Zhejiang Province People’s Republic of China

**Keywords:** Fat grafting, Cell-assisted lipotransfer, Fat survival rate, Patient satisfaction rate, Meta-analysis

## Abstract

**Background:**

Cell-assisted lipotransfer is a novel technique for fat grafting. This study aimed to investigate the clinical efficacy of cell-assisted lipotransfer technology compared with conventional fat grafting.

**Methods:**

According to PRISMA guidelines, related articles in PubMed, Embase and Cochrane library were systematically searched. Studies focusing on fat survival rate and/or patient satisfaction rate for fat grafting alone versus cell-assisted lipotransfer were retrieved. Estimated fat survival and patient satisfaction rates were pooled. Subgroup analysis was stratified by the transplant site. Publication bias was conducted. Furthermore, the stability of results was assessed by sensitivity analysis.

**Results:**

Nine articles were included in the meta-analysis. Significant heterogeneity was observed among individual studies for fat survival rate assessment (*I* ^2^ = 98.3%, *P* < 0.001). The fat survival rate was significantly higher in the cell-assisted lipotransfer group than in the control group [weighted mean difference = 25.85, 95% confidence interval 5.39–46.31; *P* = 0.013]. Notably, results remained unchanged in the sensitivity analyses. No significant difference was found in the patient satisfaction rate between the cell-assisted lipotransfer and control groups [odds ratio = 3.69, 95% confidence interval 0.73–18.53; *P* = 0.113]. In subgroup analysis, a significantly higher patient satisfaction rate was found in cell-assisted lipotransfer fat graft group in the face (odds ratio = 18.85, 95% confidence interval 9.03, 28.68; *P* < 0.001) and arm (odds ratio = 64.60, 95% confidence interval 58.79, 70.41; *P* < 0.001) than in the controls. Finally, no significant publication bias was found (*P* = 0.371).

**Conclusion:**

This study suggests that cell-assisted lipotransfer is superior to conventional lipoinjection with improved fat survival rate. However, the long-term efficacy should be evaluated in further studies.

## Background

Fat grafting was first reported in 1989, but the use of this technology was restricted due to its unpredictability and low graft survival rate [1, 2]. In 2008, a new technology based on adipose tissue-derived stromal cells (ADSCs) was reported by Yoshimura and colleagues [3]. Subsequently, cell-assisted lipotransfer (CAL) has become increasingly popular with the development of autologous fat harvesting, processing, reinjection and storage. Although autologous fat is biocompatible, nonimmunogenic and easily obtained, the fat resorption rate is found to be unstable, ranging from 20 to 80% [4]. However, the best technology for handling adipose tissue remains controversial.

Several clinical studies with favourable and unfavourable results using CAL compared with conventional lipoinjection have been reported. For example, Zhao et al. asserted that bone marrow-derived mesenchymal stem cell-assisted fat graft was more effective and safe for soft tissue than conventional fat grafting, based on high patient satisfaction and low complication rate [5]. However, Peltoniemi et al. reported similar survival rates for patients who underwent cell enrichment and water-assisted lipotransfer [6].

A meta-analysis that supported the superior clinical efficacy of CAL has been reported [7]. Subsequently, several clinical studies confirmed these findings [8, 9]. Moreover, although no study has discussed this issue, the transplant site may be one of the factors influencing the efficacy of fat grafting [10, 11]. Thus, we conducted a meta-analysis to investigate whether CAL could improve fat survival and patient satisfaction rates. We also performed a subgroup analysis stratified by the transplant site.

## Methods

### Search strategy

The meta-analysis was performed according to PRISMA guidelines. Related articles in PubMed, Embase and the Cochrane library were systematically searched with no language restriction. Articles published before 20 April 2017and containing the following terms were included in the study: (“fat graft” or “fat transplantation” or “lipotransfer” or “lipofilling” or “lipografts” or “autologous fat”) AND (“SVF” or “stem cell” or “ADSC” or “ASC” or “ADRC” or “cell-assisted” or “progenitor-enriched” or “cell-enhanced”).Additionally, to include more available research for meta-analysis, the reference lists of the included articles were also searched.

### Study selection

The eligibility of each study was independently assessed by two investigators following the inclusion criteria: (1) the study subjects were patients who had undergone soft tissue reconstruction or filling, (2) studies assessed the clinical efficacy of autologous CAL, (3) patients in the control group were treated with fat grafting alone and (4) fat survival rate and/or patient satisfaction rate were assessed in the studies.

We excluded the following studies: (1) those in which the outcomes, including fat survival and patient satisfaction rates, were not provided or could not be calculated and (2) those studies that did not involve clinical research, such as reviews, letters and conference abstracts. If the same patients were included in more than one study, the latest reference would be included in the meta-analysis.

### Data extraction

Data extraction was independently conducted by two investigators following a pre-designed extraction form. The following information would be extracted: the first author’s name, publication year, study area, follow-up period, age, BMI, sample size, intervention strategies and research outcome. The extracted information would be checked by each other after the data extraction work had been completed, and any inconsistencies would be resolved through discussion.

### Statistical analysis

Patient satisfaction and fat survival rates were transformed into estimates of the odds ratio (OR) with its 95% confidence interval (95% CI) and weighted mean difference (WMD) with its 95% CI, respectively. Cochran’s Q statistic and *I* ^2^ test were used to analyse heterogeneity among individual studies [12]. If significant heterogeneity was identified (*P* < 0.05 or *I* ^2^ > 50%), random effects model would be used to calculate the combined effect value. Otherwise, fixed effects model would be used to combine the data.

Subgroup analysis was performed upon stratification by the transplant site. Publication bias was assessed using Egger’s test. The stability of the results was also confirmed by sensitivity analysis. All statistical analyses were performed using Stata11.0 software (Stata Corporation, College Station, TX).

## Results

### Literature search

**Fig. 1:**
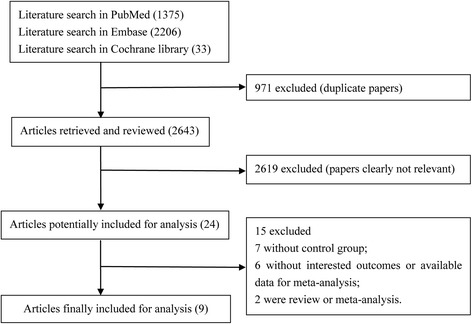
The flow chart of study selection

A flow chart of the literature search is shown in Fig. 1. According to a pre-designed selection strategy, a total of 3617 articles were originally identified in PubMed (*n* = 1375), Embase (*n* = 2206) and the Cochrane library (*n* = 36). After removing duplicated articles, 2643 articles were left. A total of 2619 of these articles were then removed after reviewing the titles and/or abstracts. After reading the full text, 15 more articles were removed, and no article was included upon a manual search. Finally, nine articles were included in the meta-analysis [5, 6, 8, 9, 13–17].

### Characteristics of included studies

The baseline characteristics of the included studies are shown in Table 1. The nine studies were reported between 2011 and 2016. The regions of the included studies widely varied, including the USA, Brazil, China, Finland and Denmark. Three transplant sites were included in the meta-analysis, which were the face, breast and arm. The follow-up period was 4–36 months. Patients in the study by Zhao et al. were treated with a bone marrow-derived mesenchymal stem cell fat graft [5], and patients in the other studies were treated with a stromal vascular fraction fat graft [6, 8, 9, 13–17]. Patients in two studies underwent two or three fat grafts [13, 16], whereas patients in the other studies underwent a single one [5, 6, 8, 9, 14, 15, 17].

**Table 1:** The baseline characteristics of included studies

Study, year, country	Recipient sites	Follow-up (months)	VMM	No. of operations	Group	Number	Age, years	BMI	Gender, M/F	FSR, %	Satisfactory
Tissiani LA, 2016, Brazil	Breast	36	MRI	1	SVF fat	11	43–58	23.5–30.9	0/11	78.8 (74.9)	NR
				1	Fat	8	36–69	20.8–32.4	0/8	51.4 (18.4)	NR
Sasaki GH, 2015, USA	Face	12	Photography	1	SVF fat	9	65.5 (52–77)	22.0 (21.0–30.8)	NR	72.9 (50.0)	NR
				1	Fat	92	60.5 (58–63)	22.0 (19.9–24.2)		38.3 (12.9)	NR
Zhao JH, 2014, China	Face	14	NA	1	BMSC fat	10	25 (20–35)	NR	3/7	NR	10
				1	Fat	26	24 (18–38)	NR	8/18	NR	24
Tanikawa DY, 2013, Brazil	Face	6	CT	1	SVF fat	7	12.1 ± 2.2	< 25	2/5	88.0 (13.0)	7
				1	Fat	7	18.7 ± 7.6	< 25	3/4	54.0 (20.0)	3
Peltoniemi HH, 2013, Finland	Breast	6	MRI	1	SVF fat	10	51 (29–58)	23.4 (20.3–32.5)	0/10	50.0 (10.0)	NR
				1	Fat	8	39 (33–63)	23.4 (20.3–25.9)	0/8	54.0 (7.0)	NR
Li J, 2013, China	Face	6	CT	1	SVF fat	26	29.5 ± 6.8	NR	0/26	64.8 (10.2)	NR
				1	Fat	12	29.1 ± 6.0	NR	0/12	46.4 (9.3)	NR
Kolle SF, 2013, Denmark	Arm	4	MRI	1	SVF fat	10	28.4 ± 8.9	24.7 ± 2.0	NR	80.9 (6.0)	NR
					Fat	10				16.3 (7.2)	NR
Chang Q, 2013, China	Face	18	CT	2 in 30%, 3 in 20%	SVF fat	10	27.5 (19–35)	NR	8/12	68.3 (1.7)	NR
					Fat	10				58.5 (1.3)	NR
Sterodimas A, 2011, Brazil	Face	18	NA	1	SVF fat	10	43.9 (22–72)	NR	3/7	NR	9
				2 in 30%, 3 in 40%	Fat	10	46.2 (25–70)		2/8	NR	9

*BMSC*, bone marrow-derived mesenchymal stem cells; *CT*, computed tomographic; *F*, female; *FSR*, fat survival rate; *M*, male; *MRI*, magnetic resonance imaging; *NR*, not reported; *NA*, not available; *SVF*, stromal vascular fraction; US, ultrasound; *VMM*, volumetric measurement method

### Meta-analysis of fat survival and patient satisfaction rates

**Fig. 2:**
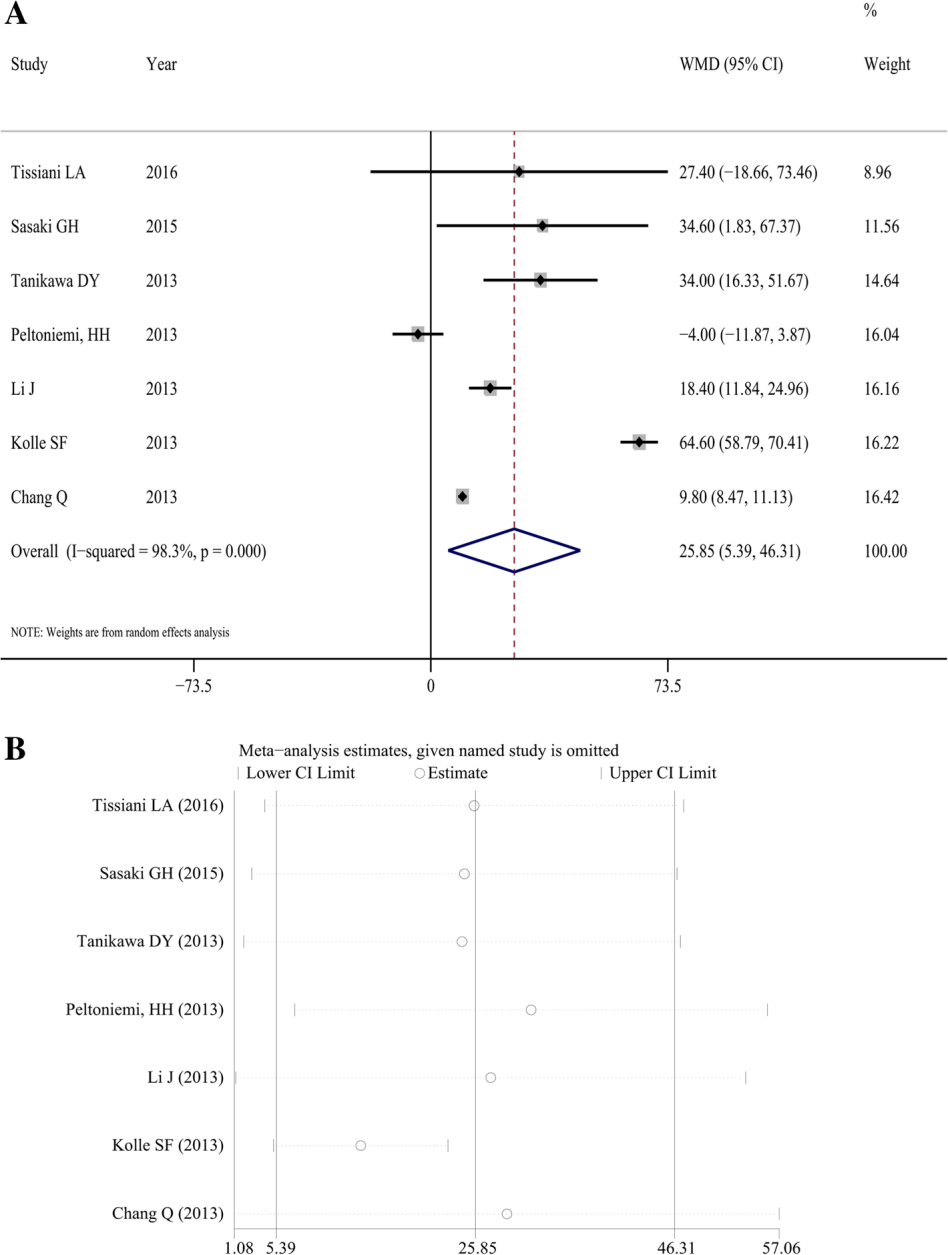
Comparison between cell-assisted lipotransfer (CAL) and conventional fat grafting regarding fat survival rate. **a** Forest map for fat survival rate comparison between CAL and conventional fat grafting. **b** Sensitivity analysis

As shown in Fig. 2a, fat survival rate was reported in seven studies [6, 8, 9, 13–15, 17]. Significant heterogeneity in this variable was observed among individual studies (*I* ^2^ = 98.3%, *P* < 0.001), and the random effects model was used to pool estimates of fat survival rate. The fat survival rate was significantly higher in the CAL group than in the control group (WMD = 25.85, 95% CI 5.39, 46.31; *P* = 0.013). The results of sensitivity analysis are shown in Fig. 2b and revealed that the results after removing each article remained unchanged, suggesting the stability of the meta-analysis.

**Fig. 3:**
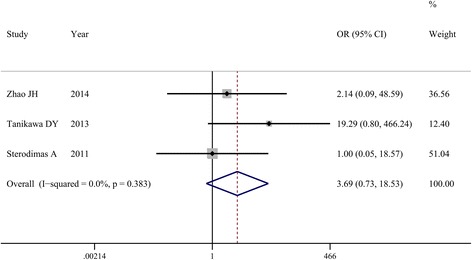
Comparison between cell-assisted lipotransfer (CAL) and conventional fat grafting regarding patient satisfaction rate

As shown in Fig. 3, patient satisfaction rate was reported in three studies [5, 16, 17]. Heterogeneity among individual studies was not statistically significant (*I* ^2^ = 0.0%, *P* = 0.383), and the fixed effects model was used to pool data on patient satisfaction rate. No significant difference was found in patient satisfaction rate between the CAL and control groups (OR = 3.69, 95% CI 0.73, 18.53; *P* = 0.113).

### Subgroup analysis

Subgroup analysis for patient satisfaction rate was stratified by the transplant site, and the results for the subgroup analysis are shown in Table 2. No significant difference was found between the CAL and control groups regarding breast fat transfer (OR = 3.16, 95% CI − 22.66, 28.99; *P* = 0.810). A significantly higher patient satisfaction rate was found in the CAL group for face fat graft than in the control group (OR = 18.85, 95% CI 9.03, 28.68; *P* < 0.001). CAL was associated with a significantly higher patient satisfaction rate for arm fat graft (OR = 64.60, 95% CI 58.79, 70.41; *P* < 0.001).

**Table 2:** Subgroup analysis for fat survival rate

Subgroup	N	WMD (95% CI)	*P* a	*P*h	*I* ^*2*^ (%)
Breast	2	3.16 (− 22.66, 28.99)	0.810	0.188	42.3
Face	4	18.85 (9.03, 28.68)	< 0.001	0.001	80.5
Arm	1	64.60 (58.79, 70.41)	< 0.001	–	–

*N* number of study, *WMD* weighted mean difference, *P* a, *P* value of association, *P*h, *P* value of heterogeneity, *CI* confidence interval

### Publication bias

Publication bias was analysed based on data for fat survival rate. No significant publication bias was found using Egger’s test (*P* = 0.371).

## Discussion

This study attempted to systematically investigate the clinical efficacy of CAL technology compared with conventional fat grafting. In total, nine articles were included in the meta-analysis. This study demonstrated that fat survival rate was significantly higher for patients using CAL. Although no significant difference was found in patient satisfaction rate between the CAL and control groups, a significantly higher patient satisfaction rate was found among patients who underwent CAL fat graft in the face and arm than in the controls. Thus, we suggest that CAL is superior to conventional lipoinjection with improved fat survival rate. However, the long-term efficacy should be evaluated in a further study.

Human adipose tissue was recommended as an ideal source of autologous cells because it is plentiful and easily obtained. Graft take rate and volume retention could be highly improved using CAL, which transforms poor-adipose derived stromal cells fat grafts into enriched ones [3]. Although the mechanism associated with fat graft survival remains unclear, lack of adequate neovascularisation has been recognised as one of the reasons for graft loss. Previous evidence supported that CAL had the ability on adipogenesis and angiogenesis in the adipose repair process [18, 19]. By pooling data from previous clinical data, we proved that CAL was superior to conventional lipoinjection with improved fat survival rate. However, the precise mechanism of CAL on improving fat survival rate should be further studied.

Although a significant improvement in fat survival rate in CAL fat enrichment was demonstrated in the meta-analysis, significant heterogeneity in this variable among the studies should not be ignored. It is known that fat survival rate is affected by the method of obtaining, isolating and preparing cells in different clinical settings [20, 21]. This would make it difficult to compare studies according to different techniques used in the surgery. Moreover, after transfer, the amount of fibrosis induced and the number of viable fat cells were reported to be the main factors for the clinical longevity of the correction [22–24]. Additionally, the survival of fat cell grafts would also be affected by the anatomic site and the mobility and vascularity of the recipient tissue [25]. Fat graft results would also depend on the background of patients, technique used and surgeon’s expertise. However, the above factors could not be fully balanced among individual studies in the meta-analysis, which may explain the significant heterogeneity. For the technology of fat grafting, further technical and outcome standardisation is thus required.

No significant difference was found between CAL and conventional fat grafting in breast fat transfer. Patients were more satisfied with CAL in the arm and face than with conventional fat grafting. No recent study has provided evidence regarding the fat transfer site that is superior. However, it should be recommended that clinical efficacy be assessed based on transplant site.

The strengths of this meta-analysis are as follows: First, the clinical efficacy of CAL or fat grafting alone was quantitatively analysed based on case-control studies. Second, although significant heterogeneity was found for data on fat survival rate, the findings of sensitivity analysis guaranteed the stability of the results. Third, no significant publication bias was found in the meta-analysis.

Some limitations should also be noted in the meta-analysis. First, only four out of nine included studies were randomised controlled trials. Although the quality of included studies was fit for the meta-analysis, the type of research design might limit the strength of the conclusion [9, 14, 16, 17]. Moreover, fat grafts were randomly injected into the posterior part of the right and left upper arms in the study by Kølle et al. [14]. Therefore, analysis of the quality of the included studies could not be performed in the meta-analysis. Second, the number of included studies was small; therefore, further study is needed to verify the current conclusion by incorporating more randomised controlled trials with high quality.

## Conclusion

Numerous methods have been proposed to enhance the survival of fat grafts, but no definitive treatment protocol is available. CAL offers new perspectives for improving fat graft survival. In summary, this study suggests that CAL is superior to conventional lipoinjection with improved fat survival rate. However, its long-term clinical efficacy should be evaluated in a further study.

## Abbreviations

95% CI: 95% confidence interval
ADSCs: Adipose tissue-derived stromal cells
CAL: Cell-assisted lipotransfer
OR: Odds ratio
WMD: Weighted mean difference
